# Flavonols reduce aortic atherosclerosis lesion area in apolipoprotein E deficient mice: A systematic review and meta-analysis

**DOI:** 10.1371/journal.pone.0181832

**Published:** 2017-07-25

**Authors:** James Phie, Smriti M. Krishna, Joseph V. Moxon, Safraz M. Omer, Robert Kinobe, Jonathan Golledge

**Affiliations:** 1 The Vascular Biology Unit, Queensland Research Centre for Peripheral Vascular Disease, School of Medicine & Dentistry, James Cook University, Townsville, Queensland, Australia; 2 College of Public Health, Medical & Veterinary Sciences, James Cook University, Townsville, Queensland, Australia; 3 Department of Vascular and Endovascular Surgery, The Townsville Hospital, Townsville, Queensland, Australia; University of Kansas Medical Center, UNITED STATES

## Abstract

Diets rich in flavonoids have been reported to have beneficial effects in the primary prevention of cardiovascular events. There are limited data, however, on the cardiovascular benefits of purified flavonoids. The aim of this systematic review and meta-analysis was to examine the reported effects of isolated flavonoids on aortic atherosclerosis in a mouse model. Medline, Pubmed, Science direct and Web of Science were searched to identify studies which examined the effect of isolated flavonoids on aortic atherosclerosis in apolipoprotein E deficient mice. A meta-analysis was performed to determine the overall effect of the flavonoids, and sub-analyses were performed to compare the effects of the flavonols and flavan-3-ols. Eleven studies, which examined a total of 208 mice receiving a flavonoid and 126 control mice, were included. Overall the flavonoids significantly reduced aortic atherosclerosis (SMD 1.10, 95% CI 0.69, 1.51). Of the 18 flavonoid interventions examined 12 were flavonols and 3 were flavan-3-ols. Sub-analyses suggested that the flavonols (SMD 1.31, 95% CI 0.66, 1.91) but not the flavan-3-ols (SMD 0.33, 95% CI -0.19, 0.85) significantly decreased atherosclerosis area. Of the eleven studies, only one examined histological markers of atherosclerosis plaque stability. Most studies did not report blinding of outcome assessors or reproducibility of the primary outcome, and did not justify the sample size used and flavonoid dose administered. Based on the included studies, the flavonols appear to be the most effective flavonoids for reducing aortic atherosclerotic lesion area in apolipoprotein E deficient mice.

## Introduction

Atherosclerosis and associated thrombosis are the main underlying cause of stroke, myocardial infarction and peripheral artery disease which, collectively, are the leading cause of death globally [1]. Much of the focus on the primary and secondary prevention of cardiovascular disease over the last few decades has been on drugs to manage cardiovascular risk factors such as statins, blood pressure lowering medications and anti-platelet agents [2–4]. Diet has been linked to the development and complications of cardiovascular disease for centuries however it is only more recently that trials demonstrating the benefits of dietary interventions in humans have been completed [5–7]. A recent multicentre randomized controlled trial, which included 7447 participants, reported that the Mediterranean diet with either extra virgin olive oil or nut supplementation reduced the incidence of cardiovascular end points by 30%, compared to control subjects educated on a low fat diet [8]. The beneficial effects of extra virgin olive oil and nuts have been linked to their high phenolic content [9, 10], and reductions in blood pressure and low density lipoprotein (LDL) oxidation have been reported for other foods high in phenols including dark chocolate, green tea, apples, tomatoes, kale, lettuce and onions [11–13].

**Fig 1:**
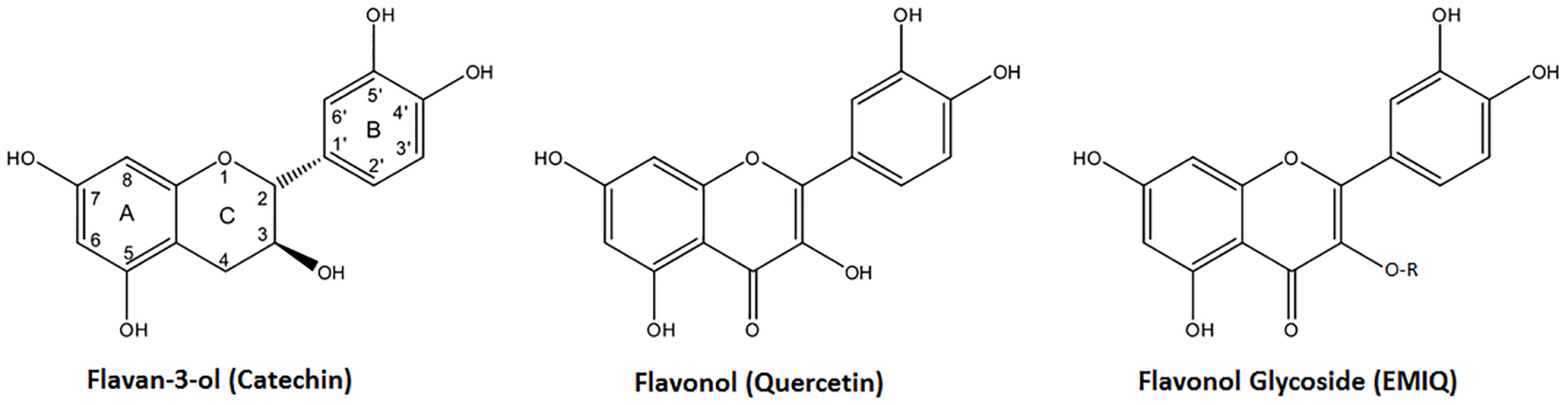
Properties of the major flavonoid classes (examples in brackets) used in atherosclerosis research in apolipoprotein E mice. Catechin and epicatechin are flavan-3-ols; Quercetin, Kaempferol and Isorhamnetin are flavonols; and EMIQ and myricitrin are flavonol glycosides. EMIQ, Enzymatically modified isoquercitrin; R, glucose chain (n = 3–9). Adapted from de Pascual-Teresa, Moreno [17].

A subclass of phenols, known as the flavonoids, have been of particular interest in recent research for their potent anti-oxidant and anti-atherosclerosis effects [14]. The most studied flavonoids are the flavan-3-ols, the flavonols, and the flavonol glycosides (e.g. enzymatically modified isoquercitrin; EMIQ) (Fig 1) [15, 16]. However, the impact of consuming purified flavonoids on cardiovascular events have not been examined in human clinical trials.

Apolipoprotein E deficient (ApoE^-/-^) mice have been used to assess the effect of flavonoids on atherosclerosis, however the overall effect of the flavonoids, and the effect of individual classes of flavonoids on the severity of atherosclerosis remain unclear. The ApoE^-/-^ mouse model is particularly useful for studying atherosclerosis due to its quick and predictable development of atherosclerosis [18, 19]. Furthermore, the model responds to lipid lowering interventions which have been shown to reduce complications of atherosclerosis in humans [18, 20, 21]. The aim of this systematic review and meta-analysis was to examine the effect of purified flavonoids on the severity of aortic atherosclerosis in ApoE^-/-^ mice.

## Materials and methods

### Search strategy, inclusion and exclusion criteria

This review was prepared according to the 2009 Preferred Reporting Items of Systematic Reviews and Meta-Analyses (PRISMA) statement. Literature searches were conducted to identify pre-clinical studies testing the effects of flavonoids on atherosclerosis lesion area in ApoE^-/-^ mice. Medline, Pubmed, Science direct and Web of Science were searched between 16^th^ March 2016 and 27^th^ May 2017. Science direct searches were filtered to include only journal articles. The full search strategy included the terms ("Apolipoprotein* E" OR ApoE) AND (Mice OR Mouse) AND (Phenol* OR Flavonoid* OR Quercetin OR Catechin OR Glabridin OR Kaempferol OR Epicatechin OR Baicalin OR Isorhamnetin) utilising both medical subject headings (MeSH) and keyword searches. Resulting titles and abstracts from all years were screened for relevance and all potentially eligible abstracts were assessed in full text by one reviewer (JP). Reference lists of included studies were also screened for relevant articles. Controlled studies which used purified flavonoids, or flavonoids with glucoside moieties such as isoquercitrin, in an ApoE^-/-^ mouse model were included. Of these, only studies which included atherosclerosis lesion area as an outcome measure were included. Studies which did not report atherosclerosis lesion area as a percentage or as lesion area out of total area measured were excluded. Included studies were restricted to those published in English. Reviews, editorials, books, letters, case reports, and clinical trials were excluded. Studies where sample size could not be determined were excluded.

### Data extraction and quality assessment tool

Full text articles eligible for data extraction were independently assessed by three authors (JP, SMK and SMO) and results were later discussed in a consensus meeting. The data extracted included mouse age, sex and diet, flavonoid purity and dose, duration of treatment, control and treatment group sample sizes, location of atherosclerosis area assessed, stain used for lesion assessment, lesion area as a percentage of total area, plasma triglyceride (TG), LDL cholesterol (LDL-C), high density lipoprotein cholesterol (HDL-C), total cholesterol (TC) and measures of LDL-oxidation and reactive oxygen species. Data were extrapolated from figures in 10 of the studies using Adobe Photoshop CS6. In cases of missing data, corresponding authors were contacted. Authors of seven papers were contacted [22–28], and two responded [25, 27]. Study quality was assessed using a quality assessment tool adapted from a previous preclinical systematic review (S1 Table) [29]. Questions focused on the reporting of study design, mouse age and sex, diet, housing conditions, method of group allocation, ethics approval, justification of sample sizes, justification of flavonoid dose, flavonoid purification processes, methods of lesion area analysis and blinding procedures for outcome assessments. Each question was assessed as a yes or no answer and the number of yes answers reported as a percentage out of 17 questions, or out of 15 questions for studies that did not measure plasma lipids. These percentage scores were used as an indication of overall study quality. Studies were rated as poor (<50%), moderate (51–75%), good (76–90%), or excellent quality (91–100%).

### Statistical analysis

A meta-analysis was used to assess the overall effects of the administration of purified flavonoids on atherosclerosis lesion area in ApoE^-/-^ mice. In four studies [30–33], the same control group was used to compare multiple flavonoids or flavonoid doses. In these instances, the number of control animals was divided by the number of comparator groups to prevent an artificial increase in sample size in the meta-analysis. Sub-analyses were performed to test the overall effect of the flavonols and flavan-3-ols on atherosclerosis lesion area.

Heterogeneity in the data was expected due to the use of multiple flavonoids, at different doses with different durations of treatment, and therefore random effects models were used. Data were expressed as standardised mean difference with 95% confidence intervals. Sensitivity analyses were performed using the leave one out approach to test the effect of each study on the standard mean difference. I^2^ values were used to assess heterogeneity between studies. Heterogeneity above 30% was considered moderate, and heterogeneity above 50% was considered substantial. Funnel plots were used to test for publication bias. Statistical analyses were performed using RevMan 5.3, and tests were considered significant when P values were <0.05.

## Results

### Study selection

**Fig 2:**
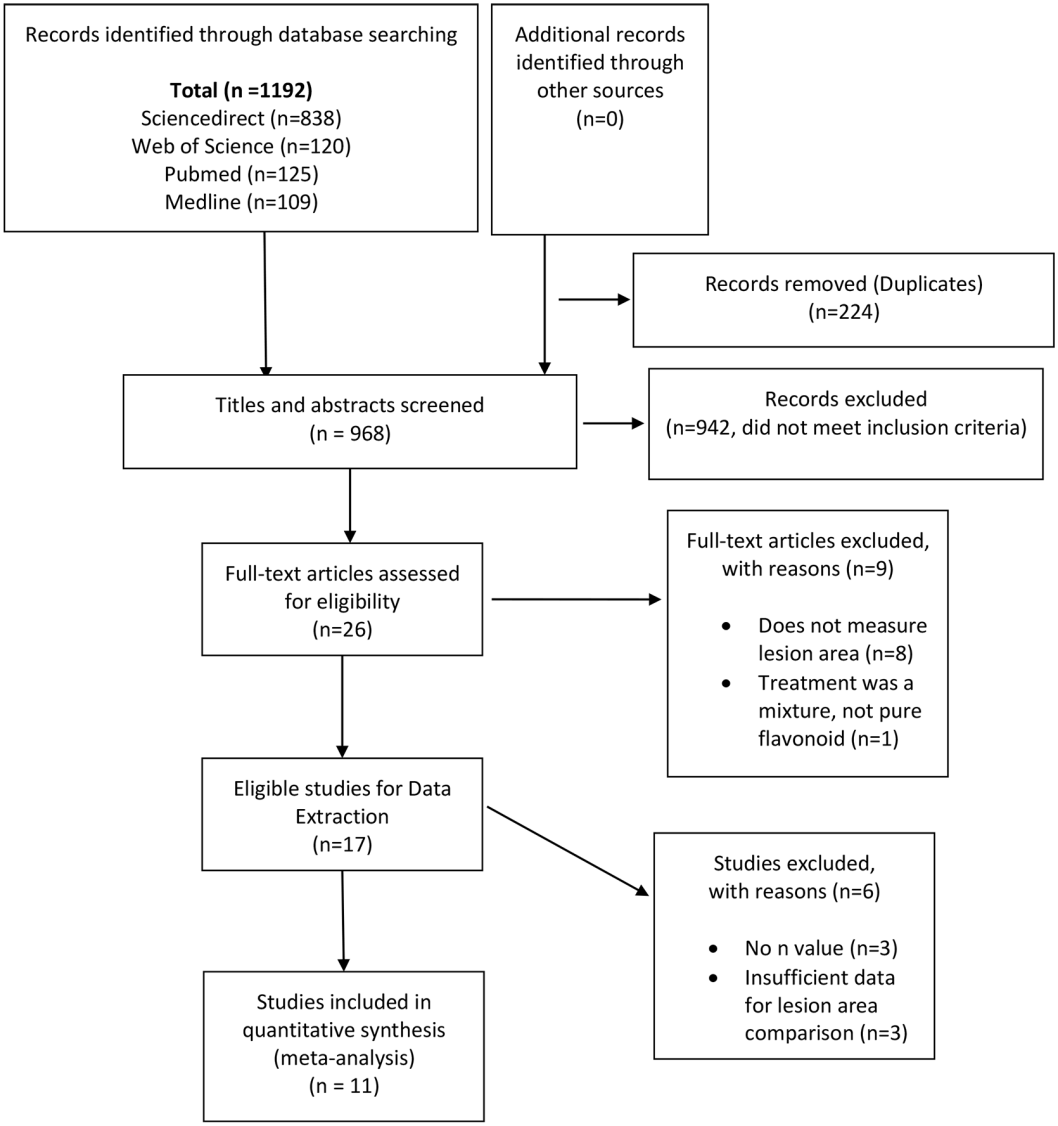
Preferred Reporting Items of Systematic Review and Meta-analyses (PRISMA) flow diagram. A total of 1192 articles were identified from Science direct, Web of Science, Pubmed and Medline. Of these, 26 full-text articles were assessed for eligibility and 11 articles were included in the review. Eight studies were excluded as they did not measure atherosclerosis lesion area. One study was excluded as it administered a crude extract. Three studies were excluded because sample sizes could not be retrieved and three studies were excluded because of insufficient data for lesion area comparison. *From*: Mohar D, Liberati A, Tetzlaff J, Altman DG, The PRISMA Group (2009). *P*referred *R*eporting *I*tems for *S*ystematic Reviews and *M*eta-*A*nalyses: The PRISMA Statement. PLOS Med 6(7): e1000097. doi:10.1371/journal.pmed1000097 **For more information, visit** www.prisma-statement.org.

Database searches identified a total of 1192 studies, of which 224 articles were duplicates (Fig 2). The titles and abstracts of the remaining 968 articles were screened and 942 were excluded. 26 full text articles were assessed. Eight of these studies were excluded because atherosclerosis lesion area was not reported and one study was excluded because a crude extract rather than a purified flavonoid was administered. After contacting corresponding authors, a further three studies were excluded because sample sizes for lesion area measurements could not be obtained [22, 23, 27], and three studies were excluded after no reply from authors because total area of the aorta measured was not reported [24, 28, 34]. In total, eleven studies were included in the current systematic review and meta-analysis.

### Study characteristics

The characteristics of the eleven included studies are shown in Table 1. Mice from all included studies were male, except for one study in which the sex was not reported [30]. Mice received a standard chow diet in three studies [25, 30, 32]; a high fat diet in seven studies containing either 20% fat and 0.3% cholesterol [35–37], 21% fat and 1.25% cholesterol [33], 47% carbohydrates, 21% fat and 20% protein [26], or 1.25% cholesterol and 10% coconut oil [38, 39]; and the American Institute of Nutrition (AIN) 93 diet, containing 4% fat, 73% carbohydrates, and 14% protein, in one study [31]. The age of the mice when flavonoid administration was initiated was four weeks in one study [30], six weeks in seven studies [25, 26, 31, 33, 35–37], eight weeks in one study [39], nine weeks in one study [38], and 15 weeks in one study [32]. Six studies administered the flavonoids via the intragastric route [32, 35–39], two studies administered the flavonoids by adding it to the drinking water [25, 30], and three studies administered the flavonoids by adding to the chow [26, 31, 33].

**Table 1:** Characteristics of studies included in this meta-analysis.

Study	Flavonoid	Purity	Mouse Age (weeks)	Mouse Sex	Diet	Study length (weeks)	Dose (mg/ kg/d)	Route	Lesion area measured	Staining	Analysis
[38]	Baicalin	98%	9	Male	HCD	12	100	IG	Aortic root	Oil red O	Cross sectional
[39]	Baicalin	98%	8	Male	HFD	12	100	IG	Aortic root	Oil red O	Cross sectional
[30]	Catechin	?	4	?	SCD	6	~1.6	Water	Aortic arch	Osmium tetroxide	Cross sectional
[31]	Epicatechin	98%	6	Male	AIN-93M	26	64	Chow	Aortic Sinus, Thoracic aorta	Sudan IV	Cross sectional
[25]	Glabridin	98%	6	Male	SCD	6	~0.6	Water	Aortic valves	Osmium	Cross sectional
[26]	EMIQ	?	6	Male	HFD	14	~30	Chow	Aortic sinus	Oil red O	Cross sectional
[35]	Isorhamnetin	98%	6	Male	HFD	8	20	IG	Aortic valves	Oil red O	Cross sectional
[32]	Kaempferol	98%	15	Male	SCD	4	50 or 100	IG	Aortic root to Iliac branches	Sudan IV	En face (longitudinal)
[37]	Myricitrin	98%	6	Male	HFD	8	50	IG	Aortic root	Oil red O	Cross sectional
[36]	Myricitrin	98%	6	Male	HFD	6	50	IG	Aortic root	Oil red O	Cross sectional
[30]	Quercetin	?	4	?	SCD	6	~1.6	Water	Aortic arch	Osmium	Cross sectional
[31]	Quercetin	98%	6	Male	AIN-93M	26	64	Chow	Aortic Sinus, Thoracic aorta	Sudan IV	Cross sectional
[33]	Quercetin	98%	6	Male	HFD	24	25–100	Chow	Aortic Sinus	H&E	Cross sectional

^**~**^ indicates dose was calculated based on average body weights and food consumption reported in previous ApoE^-/-^ mouse studies. AIN, American institute of Nutrition; EMIQ, enzymatically modified isoquercitrin; HCD, high-cholesterol diet; HFD, high-fat diet; IG, Intragastric; M, maintenance; SCD, standard chow diet;

^?^, unknown; H&E, hematoxylin and eosin.

The eleven included studies collectively measured the effects of nine unique flavonoids, at multiple doses in a total of 18 comparisons, based on the outcome of atherosclerosis lesion area. Quercetin was administered at 25, 50 and 100mg/kg/d for 24 weeks [33], 64mg/kg/d for 26 weeks [31] and 1.6mg/kg/d for six weeks [30]. Myricitrin was used in two studies at 50mg/kg/d for six weeks [37] and eight weeks [36]. Baicalin was administered at 100mg/kg/d for 12 weeks in two studies [38, 39]. Catechin was administered at 1.6mg/kg/d for six weeks [30]. Epicatechin was administered at 64mg/kg/d for 26 weeks [31]. Glabridin was given at 0.6mg/kg/d for six weeks [25]. Isorhamnetin was administered at 20mg/kg/d for 8 weeks [35]. Kaempferol was given at 50 and 100mg/kg/d for 4 weeks [32]. EMIQ was administered at 30mg/kg/d for 14 weeks [26]. Three studies did not report dose per kilogram and gave no body weight measurements [25, 26, 30], therefore we calculated an estimated dose assuming an average mouse weight of 30g per mouse [40] and an average food intake of 3.45g/d [41]. The purity of individual flavonoids was >98% in nine studies, as measured through high performance liquid chromatography [25, 31–33, 35–39]. The purification procedure was described but purity was not stated in one study [26], and no flavonoid source or purification procedure was given for one study [30].

### Atherosclerosis lesion area

Ten studies measured cross sectional aortic lesion area in mice receiving flavonoids and controls [25, 26, 30, 31, 33, 35–39]. Of these, nine of the studies measured aortic lesion area at the level of the aortic root, sinus or valves [25, 26, 31, 33, 35–39], and one study also measured lesion area within the thoracic aorta [31]. One study measured aortic lesion area only within the aortic arch [30]. One study measured longitudinal aortic lesion area from the aortic root to the iliac branches (Table 1) [32]. All studies reported that the administered flavonoid significantly reduced atherosclerosis lesion area compared with control mice, except one study testing epicatechin where no significant difference was reported (Table 2) [31].

**Table 2:** Effect of different flavonoids on atherosclerosis lesion area in ApoE^-/-^ mice and key aspects of the methods of the included studies.

Study	Flavonoid (Dose, mg/kg/d)	Lesion area decrease (%)	P<	Sample Size		Blinded	Repeated measures	Sample size justified	Dose justified
				C	E				
[38]	Baicalin (100)	9.40 ± 5.70	0.05	10	10	N	N	N	N
[39]	Baicalin (100)	19.38 ± 3.26	0.01	5	5	N	N	N	N
[30]	Catechin (1.6)	2.09 ± 1.51	0.01	20	19	Y	N	N	N
[31]	Epicatechin (64)	5.31 ± 6.34	NS	20	19	N	N	Y	Y
[31]	Epicatechin (64)	5.43 ± 8.23	NS	20	19	N	N	Y	Y
[25]	Glabridin (0.6)	30.55 ± 7.70	0.01	14	14	N	N	N	N
[26]	EMIQ (30)	7.5 ± 4.30	0.01	8	7	N	N	N	N
[35]	Isorhamnetin (20)	19.21 ± 4.75	0.01	10	10	N	N	N	N
[32]	Kaempferol (50)	4.85 ± 1.14	0.05	10	10	N	N	N	N
[32]	Kaempferol (100)	8.84 ± 1.01	0.01	10	10	N	N	N	N
[37]	Myricitrin (50)	3.96 ± 5.01	0.05	10	10	N	N	N	N
[36]	Myricitrin (50)	8.11 ± 6.11	0.01	10	10	N	N	N	N
[30]	Quercetin (1.6)	2.44 ± 1.53	0.01	20	20	Y	N	N	N
[31]	Quercetin (64)	28.5 ± 4.63	0.05	20	18	N	N	Y	Y
[31]	Quercetin (64)	18.57 ± 8.27	0.05	20	18	N	N	Y	Y
[33]	Quercetin (25)	2.09 ± 1.93	NS	3	3	N	N	N	Y
[33]	Quercetin (50)	5.77 ± 1.94	NS	3	3	N	N	N	Y
[33]	Quercetin (100)	12.21 ± 1.95	0.05	3	3	N	N	N	Y

NS, not significant; C, control; E, experimental group receiving flavonoid; Y, yes; N, no.

### Plasma lipids

Eight studies measured plasma [25, 26, 31, 35, 38] or serum [36, 37, 39] TC, six studies measured plasma [26, 35, 37] or serum TG [36, 37, 39], six studies measured plasma [30, 35, 38] or serum [36, 37, 39] LDL-C, and seven studies measured plasma [26, 30, 35, 38] or serum [36, 37, 39] HDL-C. In all studies, flavonoids were reported to have no significant effect on plasma or serum TC, TG, LDL-C or HDL-C.

### Oxidative stress

Three of the included studies measured the effects of flavonoids on indicators of oxidative stress in mouse aortic tissue, including reactive oxygen species [32], oxysterols [25], and F2 isoprostanes [31]. One study measured the effect of flavonoids on serum oxidised LDL [37]. One study investigated the effects of flavonoids on susceptibility of mouse LDL to oxidation ex vivo [30], and two studies measured oxidation in cultured cells [33, 36], with one also testing NADPH oxidase activity [33]. One study investigated human LDL oxidation in the presence of flavonoids ex vivo [25]. Quercetin [30] and glabridin [25] were reported to reduce LDL-oxidation ex vivo, while catechin was not [30]. Myricitrin was reported to significantly reduce serum LDL-oxidation in ApoE^-/-^ mice [37]. Kaempferol was reported to reduce aortic reactive oxygen species [32], and epicatechin and quercetin were reported to reduce plasma superoxide concentrations [31].

### Quality assessment of included studies

The mean quality assessment score for all eleven studies was 59% (moderate quality), with the highest score being 76% (good quality) [31] and the lowest scoring being 20% (poor quality) [25] (S1 Table). All studies failed to report intra- and inter-observer repeatability testing and blinding of the outcome assessor during lesion area analyses. All studies failed to justify the sample size and dose of flavonoids used, with the exception of one study [31]. Only one study measured the concentration of the ingested flavonoid in the plasma of mice [30]. Body weights were measured at the start and finish of the experiments in five studies [26, 31, 33, 36, 37]. Of the eleven studies, three did not confirm that study conditions were identical between the control and treatment groups [25, 30, 37]. Ethics approval statements were missing from three studies [25, 39, 42]. Two studies neglected to report the sex of the mice [25, 30], however email correspondence confirmed the sex, starting age and diet in one of these studies [25]. One study failed to report that animals were randomly allocated to control or intervention groups [25]. One study did not report the purity of the flavonoid or the process of extraction [30]. All eight studies which measured plasma lipids reported the source of assay reagents and equipment used, however reproducibility testing for plasma lipid analyses was reported for only one study [35].

### Meta-analysis, sensitivity analyses and funnel plots

**Fig 3:**
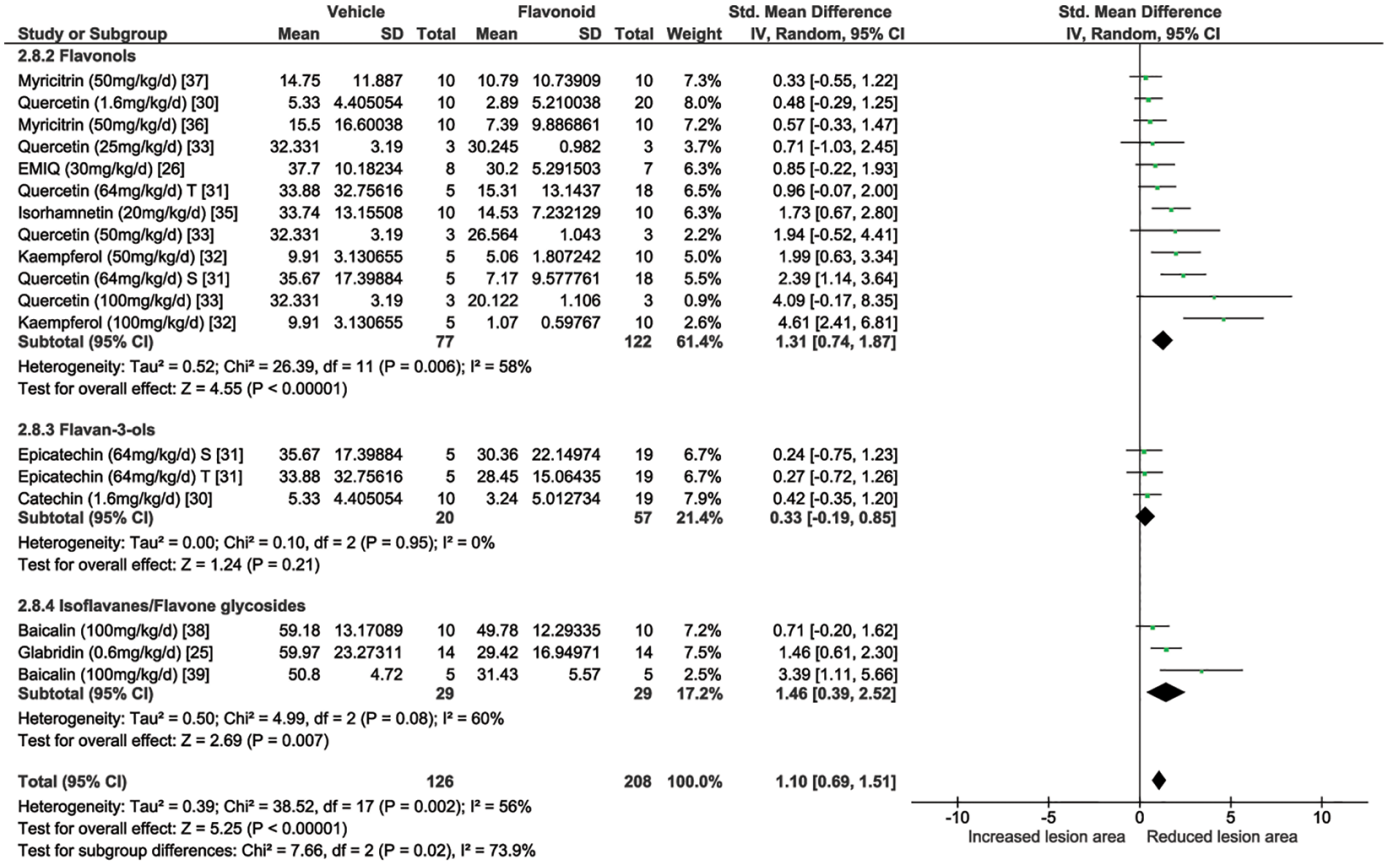
Forest plot showing the effects of flavonoids on atherosclerosis lesion area. **Sub-analyses were performed to test the effect of flavonols, flavan-3-ols, isoflavones and flavone glycosides on atherosclerosis lesion area.** The meta-analysis included 11 studies and a total of 18 flavonoid comparisons. Comparisons were made using standard mean differences and a random effects model. SD, standard deviation; CI, confidence interval; Std, standard; EMIQ, enzymatically modified isoquercitrin; S, Aortic Sinus; T, Thoracic Aorta.

Eleven studies, including a total of 18 comparisons, tested the effects of flavonoids on aortic atherosclerosis lesion area. A total of 208 flavonoid administered and 126 vehicle control ApoE^-/-^ mice were included in the overall meta-analysis (Fig 3). The overall effect of the flavonoids was to significantly lower atherosclerosis lesion area compared with controls (SMD 1.10, 95% CI 0.69, 1.51), however there was substantial heterogeneity between the studies (I^2^ = 56%). Sub-group analyses were performed to compare the effects of the flavonols and flavan-3-ols on atherosclerosis lesion area. Administration of flavonols, including flavonol glycosides, led to a significant decrease in atherosclerosis lesion area (SMD 1.31, 95% CI 0.74, 1.87) in a total of 122 experimental mice (77 control were included). The flavan-3-ols did not significantly reduce atherosclerotic lesion area (SMD 0.33, 95% CI -0.19, 0.85), based on data from a total of 57 experimental and 20 control mice.

Sensitivity analyses using the leave one out approach showed that all studies contributed to the findings of the meta-analysis and the removal of individual studies did not change the overall findings (S2 Table). Funnel plots suggested evidence of publication bias (S1 Fig).

## Discussion

The main finding of this meta-analysis was that aortic atherosclerosis lesion area was significantly lower after administration of flavonoids compared with controls in ApoE^-/-^ mice. Findings were generally consistent between studies however differences were seen in studies using flavonols compared with the flavan-3-ols. There was a high degree of heterogeneity between studies in terms of the flavonoid examined, starting age, the dose and diet administered, the duration of treatment and area of the aorta which lesion area was measured. Mice in two studies were administered standard mouse chow with experimental end points occurring at 10 [30] and 12 [25] weeks old. Previous studies suggest there is minimal plaque development by 12 weeks of age [43]. Study duration ranged from 4 weeks [32] to 26 weeks [31], and flavonoid doses ranged from 0.6mg/kg/d [25] to 100mg/kg/d [33, 38, 39]. Quality assessment revealed only two studies justified the dosage of flavonoid used [31, 33]. Heterogeneity in study methodology makes quantitative comparisons difficult for individual flavonoids and flavonoid classes. Future studies would benefit from a stronger rationale for flavonoid dose, mouse age and study duration, which are poorly justified in many current studies.

All studies failed to report reproducibility testing for lesion area measurement, and only one study reported blinding of the outcome assessor [30]. Furthermore, only one study provided sample size calculations [31]. Multiple studies did not report body weight or food consumption to allow for accurate conversion to per kilogram dosage. The funnel plot suggested the possibility of publication bias which may suggest that a number of negative studies have not been published.

Flavonoids have previously been proposed to influence atherosclerosis development through multiple mechanisms including improved lipid profile, reduction in LDL-oxidation, and reductions in a number of inflammatory cells and mediators [44]. At the doses used in the included studies, purified flavonoids were reported to have no significant effect on plasma TC, TG, LDL-C or HDL-C in ApoE^-/-^ mice. However, flavonoids were reported to have a significant effect on the oxidative stress parameters measured in all individual studies, with the exception of one study which reported that quercetin, but not catechin, reduced LDL-oxidation ex vivo [30]. Heterogeneity of methods prevented quantitative comparison of the effects of flavonoids on oxidative stress, however. Direct in-vitro antioxidant effects have been shown for multiple flavonoids [45], however there is also evidence for an indirect antioxidant effect through upregulation of endogenous heme oxygenase-1 [35, 46]. Despite the integral role of LDL-oxidation in the pathogenesis of atherosclerosis, only one of the nine included studies have measured the effects of a flavonoid on LDL-oxidation in vivo [37].

In addition to antioxidant effects, flavonoids have been reported to reduce endothelial cell apoptosis, increase aortic endothelial nitric oxide synthase activity [31, 37] and decrease dendritic cell number [38]. Flavonoids have also been reported to increase the collagen content of plaque lesions [26], and decrease expression of CD44 [32], suggesting an ability to promote atherosclerosis plaque stability [47].

The major classes of flavonoids which have been studied for their anti-atherosclerosis effects are the flavonols and the flavan-3-ols. Both of these flavonoid classes contain hydroxyl groups on carbon three of the heterocyclic ring, and are differentiated by whether or not they contain a ketone functional group on carbon 4 (Fig 1). Flavonoid glycosides were classed as flavonols due to evidence of metabolism to flavonols within the body [15, 16]. The flavonols were the most studied flavonoid, included in seven of the nine studies, while flavan-3-ols were included in two, and isoflavanes and flavone glycosides were included in only one study each. In sub-analyses limited to the flavonoid class, flavonols but not flavan-3-ols significantly reduced atherosclerosis lesion area. The small sample size of the flavan-3-ol analysis (57 experimental mice compared with 113 in the flavonol group) may possibly explain this finding. This finding may also be related to key structural differences between the flavonols and flavan-3-ols, such as the presence of a ketone functional group in the flavonols but not the flavan-3-ols. The latter may be important in reducing LDL-oxidation, which was shown to occur after administering all the flavonols tested [25, 30, 37] but not the flavan-3-ol catechin [30].

Reported beneficial effects of flavonoids on atherosclerosis severity and plaque stability suggest they may be useful for prevention and treatment of atherosclerosis in humans. A randomized crossover trial reported that quercetin, administered as quercetin-3-glycoside, significantly decreased the inflammatory biomarkers soluble endothelial selectin and interleukin 1 beta compared with placebo after four weeks supplementation [48]. However, no other clinical trials have assessed the effects of quercetin and other flavonoids on markers of atherosclerosis in humans. The antioxidant effects of the flavonoids are likely to have more impact on the formation of atherosclerosis as opposed to a decrease in size of pre-existing plaques [49]. Modifications of markers of plaque stability may therefore be a better indication of clinically relevant benefits of flavonoids in terms of likelihood of reducing cardiovascular events [50]. Currently, there is some animal research (as discussed above) but no clinical research to support a role of flavonoids in increasing plaque stability [26, 32]. Future animal studies should aim to measure the effects of flavonoids on markers of stability in pre-existing plaque lesions in order to better evaluate possible clinical outcomes.

Quercetin is currently the most studied flavonoid in animal and human studies. In the included studies, the highest dose of quercetin administered was 100mg/kg/d [33]. Previous clinical studies have administered quercetin orally at 15mg/kg/d, for up to eight weeks, without observable side effects [51]. Based on body surface area calculations, this dose translates to 185mg/kg/d in mice [52]. Therefore the doses used in these preclinical models, which were found to have beneficial effects on lesion area and atherosclerosis biomarkers, are the equivalent of relatively low doses in humans. Furthermore, quercetin is inexpensive and readily available as a dietary supplement. Based on the preclinical findings, dose tolerance, cost and availability of quercetin, future clinical studies should aim to test the benefit of quercetin supplementation in combination with currently used therapies on cardiovascular end points.

The current meta-analysis is limited by a number of factors. Firstly, none of the specific flavonoids have been tested for their effects on lesion area in more than three separate studies. Therefore we performed a statistical comparison between the most commonly studied flavonoid classes, the flavonols and flavan-3-ols, but were unable to compare individual flavonoids. Secondly, only one of the included studies measured plaque stability, and only one measured LDL oxidation in vivo, both of which are important in atherosclerosis pathology. Future animal studies should aim to assess the effects of flavonols on plaque collagen content, as preliminary evidence suggests they may improve plaque stability, which is a clinically relevant finding.

In conclusion, this meta-analysis suggests that flavonoids reduced the severity of atherosclerosis in ApoE^-/-^ mice. The flavonoid subclass, the flavonols, appear to be the most effective at reducing lesion area based on the studies available.

## Supporting information

S1 Fig Funnel plot for assessment of publication bias of included studies assessing the effects of flavonoids, flavonols and flavan-3-ols on atherosclerosis lesion area. SE, standard error; SMD, standard mean difference.
(TIF)

S1 Table Study quality assessment tool. Questions were answered with either yes, no or not applicable. Scores were expressed as a percentage of yes answers out of the total questions for each study. NA, not applicable; Y, yes; N, no, P, poor quality; M, moderate quality; G, good quality.
(DOCX)

S2 Table Leave one study out sensitivity analyses for the effects of flavonoids on atherosclerosis lesion area. SMD, standard mean difference; CI, confidence interval.
(DOCX)

S3 Table Prisma checklist for systematic reviews and meta analyses. (DOC)
