# Supplementary figures and images for: Flavonols reduce aortic atherosclerosis lesion area in apolipoprotein E deficient mice: A systematic review and meta-analysis
Source: PLoS One. 2017 Jul 25;12(7):e0181832. doi: 10.1371/journal.pone.0181832 (PMC5526572; doi:10.1371/journal.pone.0181832)

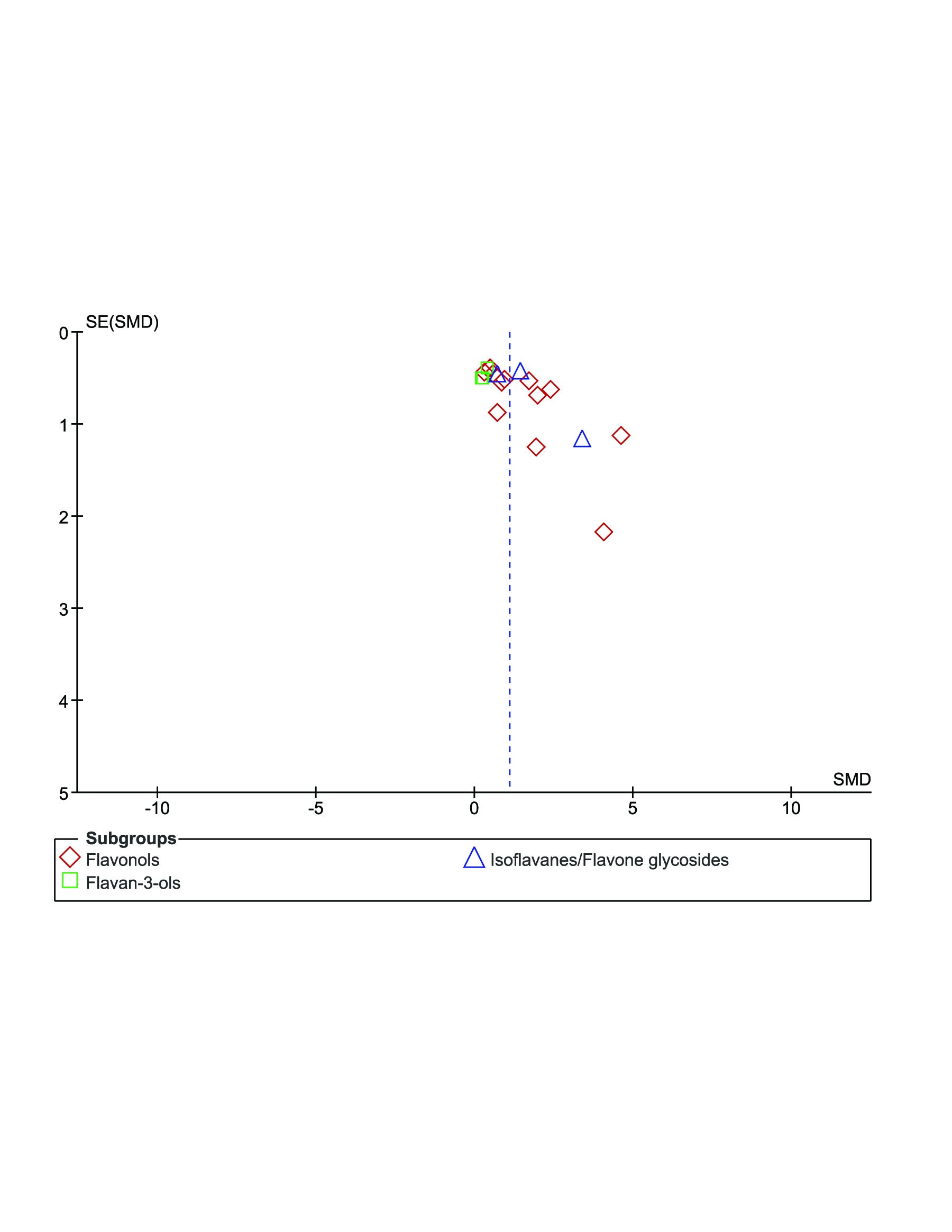

Supplement: S1 Fig — SE, standard error; SMD, standard mean difference. (TIF) [file pone.0181832.s001.tif]
