# Supplementary material for: Flavonols reduce aortic atherosclerosis lesion area in apolipoprotein E deficient mice: A systematic review and meta-analysis
Source: PLoS One. 2017 Jul 25;12(7):e0181832. doi: 10.1371/journal.pone.0181832 (PMC5526572; doi:10.1371/journal.pone.0181832)
Supplement: S1 Table — Questions were answered with either yes, no or not applicable. Scores were expressed as a percentage of yes answers out of the total questions for each study. NA, not applicable; Y, yes; N, no, P, poor quality; M, moderate quality; G, good quality. (DOCX) [file pone.0181832.s002.docx]

S1 Table

|  | **Study reference** | | | | | | | | | | |
| --- | --- | --- | --- | --- | --- | --- | --- | --- | --- | --- | --- |
| **Quality assessment questions (Part 1 – Study design and Atherosclerosis lesion area)** | 38 | 30 | 31 | 25 | 26 | 35 | 32 | 37 | 36 | 39 | 33 |
| Was there an ethics approval statement? | Y | N | Y | N | Y | Y | Y | Y | Y | N | Y |
| Were animals were randomly allocated to control/treatment groups? | Y | Y | Y | N | Y | Y | Y | Y | Y | Y | Y |
| Was the gender of mice reported? | Y | N | Y | N | Y | Y | Y | Y | Y | Y | Y |
| Was the age of mice when flavonoid administration began reported? | Y | Y | Y | N | Y | Y | Y | Y | Y | Y | Y |
| Was the diet during treatment intervention reported? | Y | Y | Y | N | Y | Y | Y | Y | Y | Y | Y |
| Was the body weight of mice measured before and after treatment? | N | N | Y | N | Y | N | N | Y | Y | N | Y |
| Was the sample size justified with statistical power analyses? | N | N | Y | N | N | N | N | N | N | N | N |
| Were study conditions identical between control and treatment groups with the exception of Flavonoid intervention? | Y | N | Y | N | Y | Y | Y | N | Y | Y | Y |
| Was the purification/extraction of the flavonoid clearly reported (or was the purity of the flavonoid stated?) | Y | N | Y | Y | Y | Y | Y | Y | Y | Y | Y |
| Was the dose of the Flavonoid justified? | N | N | Y | N | N | N | N | N | N | N | Y |
| Was the concentration of the flavonoid measured in the plasma? | N | Y | N | N | N | N | N | N | N | N | N |
| Was intra- and inter-observer repeatability testing reported for atherosclerosis lesion area analysis? | N | N | N | N | N | N | N | N | N | N | N |
| Was the method for location of section and slide preparation of section described in detail? | Y | Y | Y | Y | Y | Y | Y | Y | Y | Y | Y |
| Did the authors report the stain used for lesion area analysis? | Y | Y | Y | Y | Y | Y | Y | Y | Y | Y | Y |
| Were the investigators blinded to treatment groups during lesion area data analysis? | N | Y | N | N | N | N | N | N | N | N | N |
| **Quality assessment questions (Part 2 – Plasma lipid analysis)** | 1 | 2 | 3 | 4 | 5 | 6 | 7 | 8 | 9 | 11 | 12 |
| Was the source of assay reagents and equipment included in the methods? | Y | Y | Y | NA | Y | Y | NA | Y | Y | Y | NA |
| Was repeatability testing reported for plasma lipid analysis? | N | N | N | NA | N | Y | NA | N | N | N | NA |
| **Score (%)** | 59 | 53 | 76 | 20 | 65 | 65 | 60 | 59 | 65 | 53 | 73 |
| **Study Quality** | M | M | G | P | M | M | M | M | M | M | M |
