# Supplementary material for: Flavonols reduce aortic atherosclerosis lesion area in apolipoprotein E deficient mice: A systematic review and meta-analysis
Source: PLoS One. 2017 Jul 25;12(7):e0181832. doi: 10.1371/journal.pone.0181832 (PMC5526572; doi:10.1371/journal.pone.0181832)
Supplement: S2 Table — SMD, standard mean difference; CI, confidence interval. (DOCX) [file pone.0181832.s003.docx]

S2 Table

| **Study removed** | **SMD (95% CI)** | **P value** | **I^2^ value (%)** |
| --- | --- | --- | --- |
| None | 1.10 (0.69, 1.51) | 0.00001 | 56 |
| Liu, Liao et al. 2014 [38] | 1.14 (0.70, 1.58) | 0.00001 | 58 |
| Hayek, Fuhrman et al. 1997 [30] | 1.24 (0.77, 1.71) | 0.00001 | 57 |
| Loke, Proudfoot et al. 2010) [31] | 1.17 (0.69, 1.65) | 0.00001 | 56 |
| Rosenblat, Belinky et al. 1999 [25] | 1.08 (0.64, 1.51) | 0.00001 | 57 |
| Motoyama, Koyama et al. 2009 [26] | 1.13 (0.69, 1.56) | 0.00001 | 58 |
| Luo, Sun et al. 2015 [35] | 1.05 (0.63, 1.48) | 0.00001 | 56 |
| Xiao, Lu et al. 2011 [32] | 0.91 (0.55, 1.26) | 0.00001 | 40 |
| Qin, Luo et al. 2015 [37] | 1.16 (0.73, 1.60) | 0.00001 | 56 |
| Sun, Qin et al. 2013[36] | 1.15 (0.71, 1.59) | 0.00001 | 58 |
| Wang, Liao et al. 2016 [39] | 1.02 (0.63, 1.42) | 0.00001 | 53 |
| Xiao, Liu et al. 2017 [33] | 1.07 (0.64, 1.50) | 0.00001 | 61 |
